# Use of eye tracking to improve the identification of attention-deficit/hyperactivity disorder in children

**DOI:** 10.1038/s41598-023-41654-9

**Published:** 2023-09-02

**Authors:** Dong Yun Lee, Yunmi Shin, Rae Woong Park, Sun-Mi Cho, Sora Han, Changsoon Yoon, Jaheui Choo, Joo Min Shim, Kahee Kim, Sang-Won Jeon, Seong-Ju Kim

**Affiliations:** 1 Department of Biomedical Informatics, Ajou University School of Medicine, Suwon, Republic of Korea; 2 Department of Medical Sciences, Graduate School of Ajou University, Suwon, Republic of Korea; 3 Department of Psychiatry, Ajou University School of Medicine, Suwon, Republic of Korea; 4 Department of Biomedical Sciences, Ajou University Graduate School of Medicine, Suwon, Republic of Korea; 5 Ajou University Hospital, Suwon, Republic of Korea; 6 Department of Psychiatry, Kangbuk Samsung Hospital, Sungkyunkwan University School of Medicine, Seoul, Republic of Korea; 7 Workplace Mental Health Institute, Kangbuk Samsung Hospital, Sungkyunkwan University School of Medicine, Seoul, 04514 Republic of Korea

**Keywords:** Diagnostic markers, Eye manifestations, Translational research, ADHD

## Abstract

Attention-deficit/hyperactivity disorder (ADHD) is the most common neurodevelopmental disorder of childhood. Although it requires timely detection and intervention, existing continuous performance tests (CPTs) have limited efficacy. Research suggests that eye movement could offer important diagnostic information for ADHD. This study aimed to compare the performance of eye-tracking with that of CPTs, both alone and in combination, and to evaluate the effect of medication on eye movement and CPT outcomes. We recruited participants into an ADHD group and a healthy control group between July 2021 and March 2022 from among children aged 6–10 years (n = 30 per group). The integration of eye-tracking with CPTs produced higher values for the area under the receiver operating characteristic (AUC, 0.889) compared with using CPTs only (AUC, 0.769) for identifying patients with ADHD. The use of eye-tracking alone showed higher performance compare with the use of CPTs alone (AUC of EYE: 0.856, AUC of CPT: 0.769, *p* = 0.029). Follow-up analysis revealed that most eye-tracking and CPT indicators improved significantly after taking an ADHD medication. The use of eye movement scales could be used to differentiate children with ADHD, with the possibility that integrating eye movement scales and CPTs could improve diagnostic precision.

## Introduction

Attention-deficit/hyperactivity disorder (ADHD) is a chronic and debilitating neurodevelopmental disorder characterised by inattention, hyperactivity, and impulsivity^1^. It has a prevalence of about 5%, with symptoms persisting to adulthood in 40–60% of affected children^2, 3^. In particular, the cognitive impairment associated with ADHD can have a life-long impact^4^, affecting academic achievement, occupational attainment, and quality of life^5^.

Researchers have used various tests to evaluate cognitive function for the diagnosis of ADHD^6^. Among these, cognitive Event-Related Potentials (ERPs) in the electroencephalogram (EEG) showed robust neurophysiological differences between individuals with ADHD and without ADHD^7^. Differences in brain structural and functional measures regarding cognitive functions have been reported in patients with ADHD^8, 9^. However, despite promising results, the use of brain scanning such as brain MRI or ERPs in clinical practice is limited by its high cost and the need for technical expertise^10^. By contrast, continuous performance tests (CPTs) are relatively inexpensive and easy to use, which has resulted in their widespread use for the assessment of cognitive function in suspected ADHD^11^. However, the poor sensitivity and specificity of CPTs limit their clinical utility^12^.

Given the issues with CPTs, researchers have attempted to integrate them with other psychophysiological measures. In this regard, eye movement represents a biomarker that could offer useful information about ADHD-related cognition^13^. For example, Astar et al. showed that the integration of eye-tracking with CPTs enhanced diagnostic precision and clarified the cognitive domain in patients with ADHD^14^. Although such research has validated this approach, it has not included either children or the effect of medication. Moreover, eye movement itself could provide indirect information about learning, memory, and attention^15^. Therefore, research must also compare the performance of eye-tracking with that of CPTs.

This study aimed to fill the research gaps related to the use of eye-tracking and CPTs in children. First, we compared the performance of an eye tracker with that of CPTs. Second, we evaluated the performance of an eye tracker integrated with CPTs. Third, we evaluated the effect of medication on eye movement and CPTs.

## Methods

### Study design and participants

This study took place between July 2021 and March 2022 at the Ajou University Hospital in South Korea. Patients with ADHD and healthy controls (aged 6–10 years) were recruited through advertisements placed on bulletin boards around the hospital. The study received institutional review board approval, and all participants and caregivers provided written informed consent (no. AJIRB-MED-SUR-21-240).

We included patients in the ADHD group after a psychiatrist confirmed the diagnosis according to the criteria of the Diagnostic and Statistical Manual of Mental Disorders, Fifth Edition (DSM-5). Healthy controls were evaluated by psychiatrists for psychiatric symptoms and medical history, including ADHD. Those with a history of eye disease, autism spectrum disorder, intellectual disability, major depressive disorder, bipolar disorder, schizophrenia, Tourette syndrome, obsessive–compulsive disorder, post-traumatic stress disorder, neurological disease, or severe medical problems were excluded.

Patients with ADHD were also followed to compare symptoms by medication usage, including stimulants (methylphenidate) and non-stimulants (atomoxetine and clonidine). Among children who already used medication, primary testing took place after stopping the drug for at least one week, whereas all other children underwent primary testing before they started the drug. Follow-up testing took place 1 month after starting or re-starting ADHD medications.

### Study procedure

Participants and caregivers completed a demographic/health questionnaire and Korean versions of the ADHD Rating Scale (ARS) and Child Behavior Checklist (CBCL). To minimise external distractions, participants were then moved to a separate room with a computer, where they underwent calibration for the eye tracker and comprehensive attention testing (CAT) while their eye movements were tracked. Given the potential effect of medication on ADHD symptoms, together with the possibility of fatigue, all testing took place in the morning or early afternoon. We repeated the CAT once during follow-up after patients had received medical treatment for ADHD.

### Assessment tools

#### Attention and psychopathology

Attention was evaluated with the ARS, an 18-item scale developed by DuPaul (1991) for use with children^16^. Symptoms are rated on 4-point Likert-type scales, ranging from 0 (never) to 3 (very often). The Korean version of ARS has internal consistency ranging from 0.77 to 0.89 and test–retest reliability of 0.85^17^.

Psychopathology was assessed with the CBCL, which contains 120 behavioural items that parents rate on 3-point Likert-type scales from zero to two (Not True to Very True/Often True). Items are summed to yield a syndrome scale score across three dimensions (internalising, externalising, and total behavioural problems) and six DSM-oriented scale scores. The syndrome and DSM-oriented scales have been validated^18^, and the Korean version of the CBCL was standardised in 1997^19^.

#### Computerised CAT

Computerized CAT is the type of the computer-based CPT, and has been developed for ages 4–49^20^. The CAT is composed of six subtests: the simple selective attention (visual and auditory), continuous inhibition, interference selection, divided attention, and working memory tests. However, we excluded the auditory test for simple selective attention to allow comparison with the eye-tracking test, together with the divided attention and working memory tests that are only used from age 9 years. The CAT was performed using a computer, with participant understanding of text and voice guides presented at the start of each subtest checked by trained researchers.

Overall, the amended CAT took approximately 25 min to complete, including the assessments of selective visual attention (300 stimuli, 10 min), continuous inhibition (300 stimuli, 10 min), and interference selection (150 stimuli, 5 min). For the selective attention test, participants press the space bar button quickly when they see a circle figure at the center of a monitor. For the continuous inhibition test, they press the space bar when they see any figure except an X at the center of monitor. For the interference selection test, participants are instructed to pay attention to a central target while ignoring interference stimuli. Each subtest has five indicators: commission errors (CE), for the number of wrong responses; omission errors (OE), for the number of missed responses; mean reaction time (RT mean), for the average response time to the stimuli; standard deviation of reaction time (RT SD), for response time variability; and sensitivity coefficient (d′), for how successfully the target stimuli are differentiated from the non-target stimuli. Because only four indicators were calculated in eye-tracking, we excluded d′ from the comparison.

#### Eye-tracking apparatus and eye movement measures

Stimuli were presented on a Samsung Notebook (NT551XCJF-COM) with a 15.6-inch display, a screen resolution of 1920 × 1080 pixels, and an eye-to-screen viewing distance of approximately 50 cm. The eye-tracking apparatus (Happymind Inc. CAT test) included a host PC that tracked and computed the participant’s gaze position, as well as a display PC to present the stimuli. After downloading and running the eye-tracking programme (SeeSo; https://visual.camp/demo-archive/), eye movements were recorded at a 30 Hz sampling rate with an approximate accuracy of 1.7° (VisualCamp Co., Ltd, Seoul, Korea). Calibration to each participant in SeeSo used a five-point procedure. Online Supplementary Fig. S1 shows the graphical user interface and gaze coordinate of the eye-tracking programme.

To compare the extent of visual attention directed to the task and irrelevant regions, the participants’ field of view was divided into central and peripheral areas of interest (AOIs). As shown in online Supplementary Fig. S2, the central AOI represented the middle third of the width and length. Each subtest had four indicators: fixation ratio (FR), for the ratio of gaze fixation; mean fixation time (FT), for the average gaze fixation time to the screen; central gaze ratio (CR), for the central AOI gaze ratio; and standard deviation of gaze coordinates (gaze SD), for gaze variability. The equations used are presented in online Supplementary Fig. S3.

### Sample size

NCSS PASS (version 14) was used for the sample size calculation^21^. A recent study of eye-tracking among patients with ADHD showed that the ratio of center gaze duration between patients with ADHD (80.48%) and a healthy control group (88.35%) differed significantly according to Welch’s unequal variance *t*-test^14^. Therefore, allowing for a 5% probability of a type 1 error and a power of 80%, the minimum sample size was 29 participants in each group. Considering drop out, we decided a total sample size of 30 in each group.

### Statistical analysis

We compared baseline characteristics, ARS, CBCL, CAT indicators, and eye-tracking indicators between the ADHD group before medications and the control group by independent-sample *t*-tests and chi-square analyses for parametric and non-parametric variables, respectively. Welch’s unequal variance *t*-test was used when data failed to meet the assumption of variance homogeneity. Group differences in gaze were visualised using the gaze coordinates in subtests.

Pearson’s correlation between CAT and eye-tracking indicators was evaluated before performing the regression analyses. Using the correlation matrix, we considered that indicators with r-values of > 0.7 had multicollinearity^22^, which we evaluated further based on a variance inflation factor (VIF) of < 5^23^. Logistic regression then assessed the ability of the CAT indicators, eye-tracking indicators, or both indicators combined to identify group membership (control or ADHD). Sensitivity, specificity, and area under the curve (AUC) were compared against patients with ADHD by receiver operator characteristic (ROC) curve analysis. The method reported by DeLong et al. was used to compare AUC values^24^.

In the secondary analysis, we used paired t-tests to assess the change in ARS, CBCL, and indicators (CAT and eye-tracking) within the medication group from before to after taking medication. Differences in gaze from before to after taking medication were visualised by using gaze coordinates according to subtests.

Statistical significance was evaluated at the 5% significance level (*p* < 0.05), and all analyses were performed using R (version 4.1.0) and its open-source statistical packages.

### Ethical approval

This study was approved by the Ajou University Hospital Institutional Review Board (AJIRB-MED-SUR-21-240), and All participants and caregivers provided written informed consent. All the experiment protocol for involving human data was in accordance with the guidelines of Declaration of Helsinki.

## Results

### Participants and baseline characteristics

**Figure 1:**
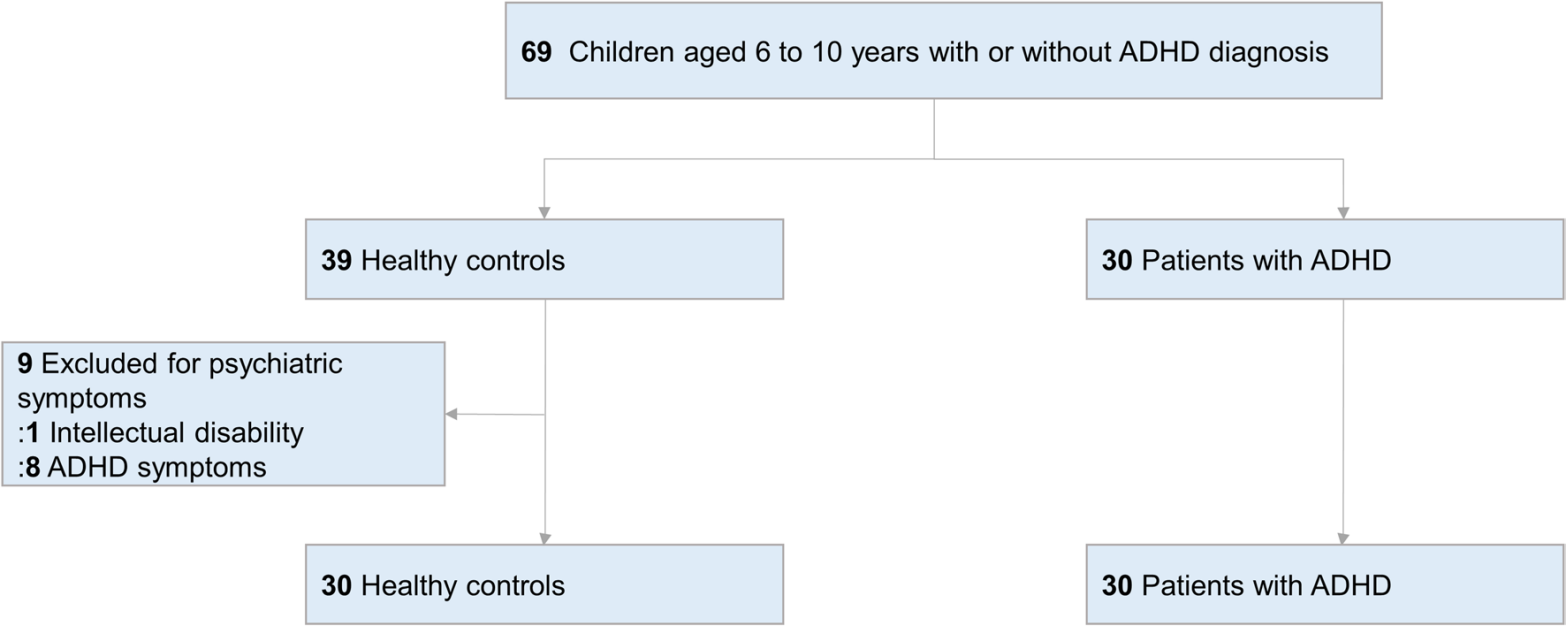
Study flowchart of children aged 6–10 years with or without ADHD.

In total, we included 30 children with a diagnosis of ADHD and 39 healthy controls, before excluding 9 participants from the control group (Fig. 1). Among patients with ADHD, 16 of the 30 (53%) were combined type, 11 (37%) were inattentive type, and 3 (10%) were hyperactive type. Of the 30 patients with ADHD, 21 (70%) reported use of ADHD medications and 9 (30%) reported no ADHD medications. 9 patients also take ADHD medications for follow-up analysis, resulting in follow-up data for 30 patients [n = 26 with stimulant medication only (methylphenidate), n = 3 with non-stimulant medication only (atomoxetine), and n = 1 with combined medication (methylphenidate and clonidine)]. Among 21 patients who stopped their medication for the experiment, 3 of them complained of irritability. The physician explained the symptoms to the patients and their caregivers, and the symptoms disappeared after the patients restarted their medication.

The ADHD and control groups did not differ significantly by age, sex, height, weight, or main caregiver (Table 1). However, the ADHD group had lower parental education (*p* = 0.002 in maternal education, *p* < 0.001 in paternal education, respectively).

**Table 1:** Descriptive statistics and statistical analyses for ADHD patients and healthy controls.

Measures	ADHD (n = 30)	Healthy control (n = 30)	*P* value
Age (mean ± *SD*)	8.0 ± 1.4	8.1 ± 1.3	0.778
Sex (*n*, (%))			0.119
Male	20 (66.7)	13 (43.3)	
Female	10 (33.3)	17 (56.7)	
Height (mean ± *SD*)	129.9 ± 8.2	130.8 ± 12.9	0.763
Weight (mean ± *SD*)	30.8 ± 8.4	30.0 ± 9.3	0.748
Main caregiver (*n*, (%))			0.189
Parents	21 (70.0)	25 (83.3)	
Grandparents	6 (20.0)	5 (16.7)	
Others	3 (10.0)	0 (0.0)	
Maternal education (*n*, (%))			0.002
College or above	20 (66.7)	30 (100.0)	
High school or below	10 (23.3)	0 (0.0)	
Paternal education (*n*, (%))			< 0.001
College or above	21 (70.0)	30 (100.0)	
High school or below	9 (30.0)	0 (0.0)	
ARS scores (mean ± *SD*)			
Inattentive scores	13.2 ± 7.3	4.1 ± 3.6	< 0.001
Hyperactivity scores	11.7 ± 7.6	2.8 ± 2.9	< 0.001
Total scores	25.8 ± 13.4	6.9 ± 5.9	< 0.001
CBCL (mean ± *SD*)			
Syndrome scales (T-score)			
Internalizing scores	62.3 ± 12.3	50.5 ± 10.8	< 0.001
Externalizing scores	66.1 ± 11.2	46.3 ± 9.5	< 0.001
Total scores	67.5 ± 11.4	48.4 ± 10.1	< 0.001
DSM 5-oriented scales (T-score)			
Affective problems	62.2 ± 9.8	53.2 ± 4.5	< 0.001
Anxiety problems	64.1 ± 11.9	55.6 ± 9.9	0.004
Somatic problems	54.1 ± 10.6	53.9 ± 5.5	0.939
ADHD	69.0 ± 15.5	53.3 ± 5.1	< 0.001
Oppositional defiant problems	63.0 ± 9.7	52.5 ± 5.3	< 0.001
Conduct problems	60.8 ± 9.5	52.1 ± 4.4	< 0.001
CAT (mean ± *SD*)			
Simple visual OE	7.0 ± 9.2	1.7 ± 3.2	0.005
Simple visual CE	13.7 ± 12.5	7.3 ± 7.8	0.022
Simple visual RT mean (ms)	547.5 ± 121.2	495.7 ± 118.4	0.100
Simple visual RT sd	235.8 ± 112.8	127.1 ± 60.6	< 0.001
Continuous inhibition OE	29.3 ± 30.1	27.1 ± 45.8	0.824
Continuous inhibition CE	24.2 ± 14.7	18.2 ± 12.6	0.095
Continuous inhibition RT mean (ms)	639.8 ± 140.9	576.2 ± 98.7	0.048
Continuous inhibition RT sd	289.9 ± 105.4	218.5 ± 102.6	0.010
Interference selection OE	23.4 ± 25.1	17.5 ± 31.4	0.427
Interference selection CE	25.2 ± 16.3	18.2 ± 17.6	0.117
Interference selection RT mean (ms)	717.7 ± 184.8	677.3 ± 195.9	0.418
Interference selection RT sd	281.7 ± 118.0	218.5 ± 116.7	0.043
Eye-tracking (mean ± *SD*)			
Simple visual FR (%)	61.4 ± 19.0	79.4 ± 16.4	< 0.001
Simple visual FT (ms)	308.5 ± 116.8	695.0 ± 612.1	0.002
Simple visual CR (%)	45.4 ± 24.0	74.9 ± 22.7	< 0.001
Simple visual Gaze sd	670.2 ± 2079.1	181.6 ± 95.4	0.209
Continuous inhibition FR (%)	57.0 ± 17.7	75.7 ± 20.9	< 0.001
Continuous inhibition FT (ms)	278.4 ± 129.8	593.2 ± 738.0	0.028
Continuous inhibition CR (%)	39.4 ± 25.2	65.4 ± 28.1	< 0.001
Continuous inhibition gaze sd	850.6 ± 2994.8	203.8 ± 121.4	0.247
Interference selection FR (%)	58.7 ± 18.8	79.9 ± 17.3	< 0.001
Interference selection FT (ms)	277.6 ± 124.1	569.5 ± 548.5	0.009
Interference selection CR (%)	47.9 ± 22.8	69.9 ± 24.8	0.001
Interference selection gaze sd	353.6 ± 342.4	204.4 ± 94.6	0.028

ARS, attention-deficit hyperactivity disorder (ADHD) rating scale; CBCL, Child Behavior Checklist; CAT, comprehensive attention test; OE, omission errors; CE, commission errors; RT mean, mean reaction time; RT sd, standard deviation of reaction time; FR, fixation ratio; FT, mean fixation time; CR, central gaze ratio; Gaze sd, standard deviation of gaze coordinates.

### Comparisons of assessments between ADHD patients and healthy controls

Table 1 shows that patients with ADHD had increased scores on the ARS overall (*p* < 0.001) and both the inattentive (*p* < 0.001) and hyperactivity (*p* < 0.001) domains. Patients with ADHD also had significantly higher scores on the syndrome and DSM-5 domains (except for somatic problems) of the CBCL. Concerning the CAT indicators, patients with ADHD performed worse than healthy controls in simple selective attention OE (*p* = 0.005), simple selective attention CE (*p* = 0.022), simple selective attention RT SD (*p* < 0.001), continuous inhibition RT mean (*p* = 0.048), continuous inhibition RT SD (*p* = 0.010), and interference selection RT SD (*p* = 0.043). Although not included in the logistic regression, there were also differences in dʹ (online Supplementary Table S1). Patients with ADHD performed worse than healthy controls in simple selective attention dʹ (*p* = 0.006) and continuous inhibition dʹ (*p* = 0.012).

Significant group differences existed for most eye-tracking indicators. Compared with controls, the ADHD group showed less fixation in simple selective attention (ratio, *p* < 0.001; time *p* = 0.002), continuous inhibition (ratio, *p* < 0.001; time, *p* = 0.028), and interference selection (ratio, *p* < 0.001; time, *p* = 0.009). The ADHD group also showed less central gaze in simple selective attention (*p* < 0.001), continuous inhibition (*p* < 0.001), and interference selection (*p* = 0.001). Moreover, patients with ADHD had increased gaze variability in the interference selection test (*p* = 0.028).

**Figure 2:**
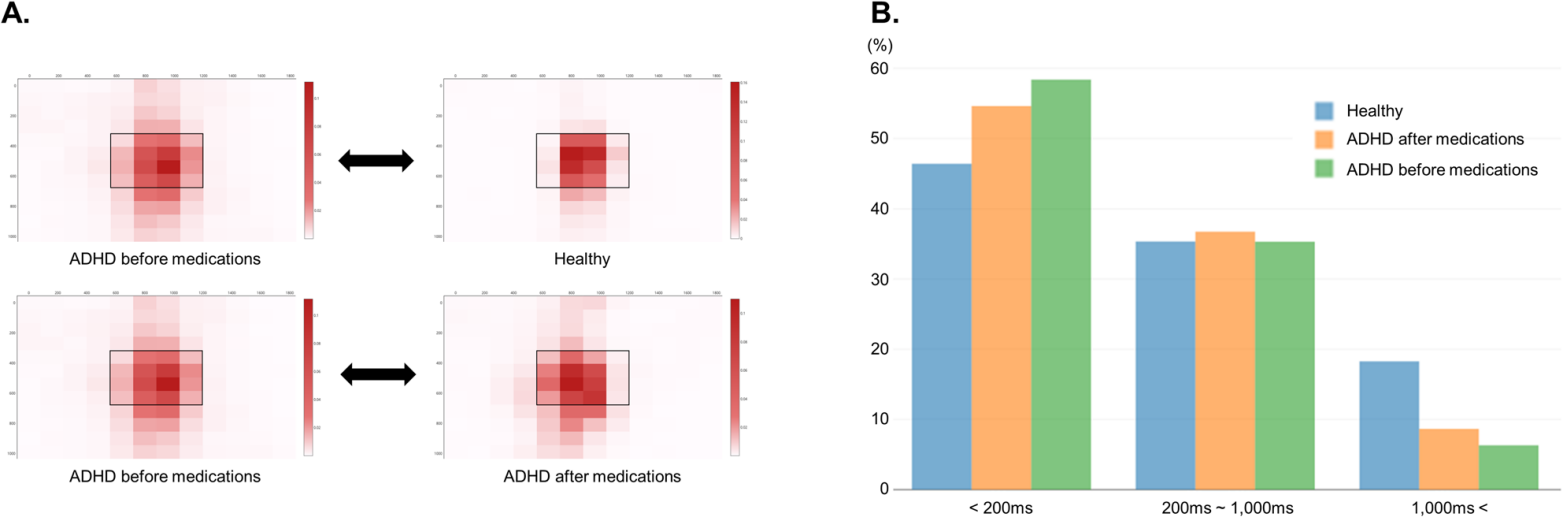
Comparison of gaze distribution in the selective attention test between the ADHD and control groups and within the ADHD group with or without medication. (**A**) Distribution of heat maps for gaze distribution. (**B**) Distribution of gaze fixation times. ADHD, attention-deficit/hyperactivity disorder.

Figure 2 presents the group differences in gaze and fixation time between the study groups for the simple selective attention test. Compared with the control group, patients with ADHD demonstrated less central gaze and shorter fixation times. The other subtests revealed similar patterns between these groups (online Supplementary Fig. S4 and S5).

### Identification of patients with ADHD

Among the CAT indicators, five were excluded due to multicollinearity and the seven remaining indicators (i.e., simple selective attention OE, simple selective attention CE, simple selective attention RT mean, continuous inhibition CE, continuous inhibition RT sd, interference selection OE, and interference selection RT sd remained) had VIF values of < 5. Among the eye-tracking indicators, eight were excluded due to multicollinearity and the four remaining indicators (i.e., simple selective attention FR, simple selective attention gaze SD, continuous inhibition FT, and interference selection CR remained) had VIF values of < 5. These results are summarised in online Supplementary Table S2 and S3.

**Figure 3:**
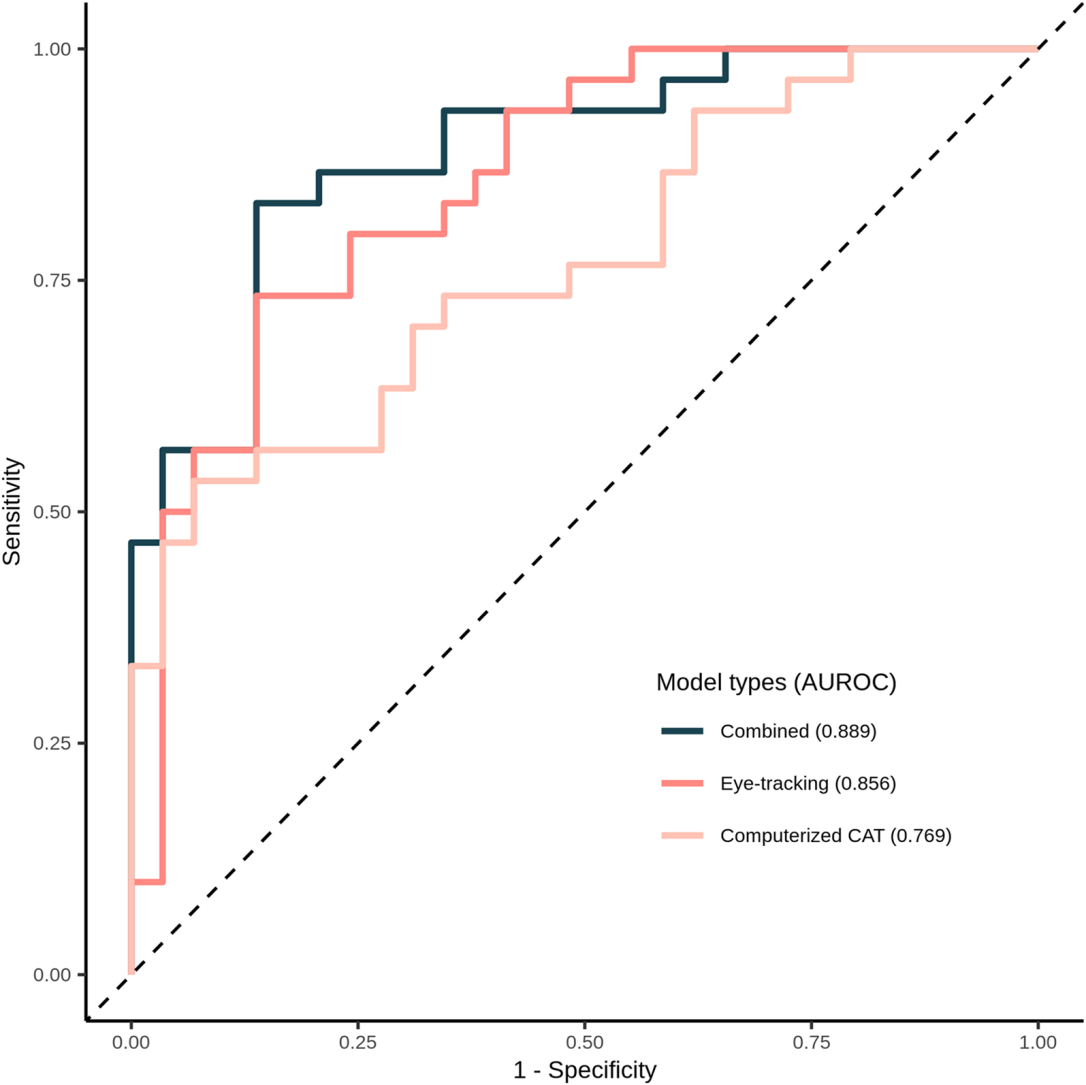
ROC curves for models identifying patients with ADHD. The AUCs for computerised CAT, eye-tracking, and both combined are compared to assess performance. ADHD attention-deficit/hyperactivity disorder; AUC, area under the receiver operating characteristic curve; CAT, comprehensive attention test; ROC, receiver operating characteristic.

Logistic regression using the CAT indicators showed a high specificity (0.931) and AUC (0.769), but a low sensitivity (0.533) (Table 2, Fig. 3, and online Supplementary Table S4). By contrast, logistic regression with the eye-tracking indicators showed high sensitivity (0.733), specificity (0.861), and AUC (0.856) values. Finally, logistic regression using both indicators revealed a sensitivity of 0.833, with a specificity of 0.862 and an AUC of 0.889. Significant differences did exist between the CAT indicators alone and eye-tracking indicators alone (*p* = 0.029) and between the CAT indicators alone and both indicator sets combined (*p* < 0.001).

**Table 2:** Results of identification of patients with ADHD.

Identification criteria	Sensitivity	Specificity	AUC
Computerized CAT	0.533	0.931	0.769
Eye-tracking	0.733	0.861	0.856
Combined (CAT + Eye tracking)	0.833	0.862	0.889

AUC, area under the receiver operating characteristic curve.

### Comparisons of assessments within ADHD patients from before to after taking medication

Treatment with ADHD medications was associated with an overall improvement in the ARS and both the CAT and eye-tracking indicators (Table 3). The ARS total (*p* = 0.019) and hyperactivity (*p* = 0.025) scores decreased significantly within the medication group from before to after taking medication. Significant improvements were from before to after taking medication for all CAT indicators (except simple selective attention OE, continuous inhibition CE, and interference selection CE) and all eye-tracking indicators (except simple selective attention gaze SD and continuous inhibition gaze SD). However, we observed no significant change in CBCL scores within the medication group from before to after taking medication.

**Table 3:** Comparisons of statistical analyses within the medication group from before to after taking medication.

Measures	After medication (n = 30)	Before medication (n = 30)	*P* value
ARS scores (mean ± *SD*)			
Inattentive scores	9.9 ± 5.8	13.2 ± 7.3	0.055
Hyperactivity scores	7.7 ± 5.5	11.7 ± 7.6	0.025
Total scores	18.2 ± 10.0	25.8 ± 13.4	0.019
CBCL (mean ± *SD*)			
Syndrome scales (T-score)			
Internalizing scores	64.3 ± 12.7	62.3 ± 12.3	0.550
Externalizing scores	64.6 ± 11.8	66.1 ± 11.2	0.608
Total scores	67.5 ± 10.7	67.5 ± 11.4	0.991
DSM 5-oriented scales (T-score)			
Affective problems	63.0 ± 9.3	62.2 ± 9.8	0.757
Anxiety problems	64.3 ± 10.0	64.1 ± 11.9	0.944
Somatic problems	55.3 ± 8.4	54.1 ± 10.6	0.618
ADHD	68.2 ± 12.9	69.0 ± 15.5	0.829
Oppositional defiant problems	63.2 ± 10.2	63.0 ± 9.7	0.928
Conduct problems	59.8 ± 8.3	60.8 ± 9.5	0.657
CAT (mean ± *SD*)			
Simple visual OE	5.0 ± 8.3	7.0 ± 9.2	0.097
Simple visual CE	9.4 ± 9.9	13.7 ± 12.5	0.030
Simple visual RT mean	514.0 ± 106.8	547.5 ± 121.1	0.014
Simple visual RT sd	186.2 ± 85.0	235.8 ± 112.8	0.002
Continuous inhibition OE	19.7 ± 26.7	29.3 ± 30.1	0.028
Continuous inhibition CE	24.7 ± 13.8	24.2 ± 14.7	0.596
Continuous inhibition RT mean	581.9 ± 117.2	639.8 ± 140.9	< 0.001
Continuous inhibition RT sd	241.2 ± 115.6	289.9 ± 105.4	< 0.001
Interference selection OE	13.7 ± 14.8	23.4 ± 25.1	0.003
Interference selection CE	25.1 ± 19.3	25.2 ± 16.3	0.485
Interference selection RT mean	660.3 ± 174.2	717.7 ± 184.8	0.003
Interference selection RT sd	236.6 ± 103.8	281.7 ± 118.0	< 0.001
Eye-tracking (mean ± *SD*)			
Simple visual FR (%)	72.4 ± 24.0	60.1 ± 19.0	0.025
Simple visual FT	545.8 ± 522.3	299.1 ± 115.1	0.009
Simple visual CR (%)	58.5 ± 28.7	43.0 ± 22.8	0.021
Simple visual Gaze sd	216.7 ± 118.2	704.3 ± 2150.4	0.121
Continuous inhibition FR (%)	68.7 ± 22.1	55.9 ± 17.8	0.022
Continuous inhibition FT	472.6 ± 492.2	274.6 ± 132.1	0.006
Continuous inhibition CR (%)	54.2 ± 24.7	38.2 ± 25.4	0.015
Continuous inhibition gaze sd	257.8 ± 114.2	898.2 ± 3098.0	0.142
Interference selection FR (%)	66.6 ± 18.5	57.2 ± 18.5	0.022
Interference selection FT	418.1 ± 321.3	267.9 ± 118.0	0.009
Interference selection CR (%)	59.6 ± 23.8	45.5 ± 21.7	0.015
Interference selection gaze sd	247.5 ± 104.7	369.2 ± 349.5	0.044

ARS, attention-deficit hyperactivity disorder (ADHD) rating scale; CBCL, Child Behavior Checklist; CAT, comprehensive attention test; OE, omission errors; CE, commission errors; RT mean, mean reaction time; RT sd, standard deviation of reaction time; FR, fixation ratio; FT, mean fixation time; CR, central gaze ratio; Gaze sd, standard deviation of gaze coordinates.

Similar to the results of the primary analysis, we also found differences in the simple selective attention test (gaze and fixation time) within the medication group from before to after taking medication. Other subtests showed similar patterns (Fig. 1, online Supplementary Fig. S4 and S5), but the differences between groups were less obvious than in the primary analyses.

## Discussion

Integrating eye-tracking with CPTs improved task performance at identifying ADHD compared with the use of CPTs alone. The use of eye-tracking alone also showed higher performance compare with the use of CPTs alone. Moreover, most eye-tracking indicators (e.g., FR and time, gaze ratio at the center, and gaze variability) differed significantly between the ADHD and control groups. Follow-up analysis of the effect of medication revealed that most eye-tracking and CPT indicators improved significantly with treatment.

Despite the limited research on eye-tracking applications in patients with ADHD, existing studies have shown the potential for them to discriminate between patients with ADHD and healthy controls^14^. For example, Elbaum et al. reported that eye-tracking had performance comparable to that of CPTs^25^, consistent with our results that the AUCs for eye-tracking was significantly higher than that of CPTs. In addition, while CPTs had a low sensitivity and a high false negative rate (47%), eye-tracking had a relatively high sensitivity and a low false negative rate (26%).

Furthermore, our findings are consistent with those of a study with a similar methodology that demonstrated improved discriminatory performance after the integration of eye-tracking^14^. When estimating central gaze duration with and without a distractor, we also showed that patients with ADHD had a lower CR than healthy controls. Estimating central gaze in this way could show the distractibility that underpins higher-order deficits in ADHD^26^. Another study revealed that evaluating eye movement distractibility could be used as a diagnostic tool for ADHD^27^. We used different indexes to estimate distractibility (e.g., fixation time, FR, and gaze variability) and show that patients with ADHD often had lower fixation ratios and times. Elsewhere, Caldani et al. also demonstrated poor fixation in youths with ADHD compared with healthy controls^28^. Moreover, our finding of increased gaze variability in patients with ADHD is consistent with previous research showing that ADHD was associated with difficulties in suppressing exploratory saccades compared with healthy controls^28, 29^. These findings are in line with the concept that children with ADHD exhibit poor inhibitory control. However, our approach produced superior differentiating performance (AUC, 0.889) than the previous study that used central gaze duration only (AUC, 0.826)^14^, possibly due the inclusion of additional distractibility variables. Overall, our findings confirmed that using eye movement indicators, alone or in combination, have the potential to improve ADHD case identification.

Analyzing the effect of medication during the follow-up among patients with ADHD produced less dramatic results than the comparisons with healthy controls. However, we did observe a significant difference in most eye-tracking variables by the presence of drug treatment. These results suggest that eye-tracking may be suitable for both diagnosing ADHD and identifying treatment response to medications.

A distinct advantage of our methods is the simplicity of the technical setup, which only required the downloading of eye-tracking software to a laptop computer. This could improve the translation of our findings to clinical practice and other settings. Eye-tracking systems could also be used in brief sessions to assess different tasks and treatment effects^15^. However, important limitations warrant further consideration.

First, we included participants aged 6–10 years old. Although research has indicated that ADHD symptoms differed significantly between these ages^30, 31^, other research has considered ages 6 to 10 years as the same age group^32^. Complementing this, we found no age differences between the ADHD and control groups. Second, this study used the relatively small sample size. However, our study included an appropriate sample size considering the previous study. Third, we did not evaluate other methods that can be integrated with eye-tracking. For example, Stolicyn et al. combined measures of eye and face movement during cognitive performance to predict depression symptoms^33^, whereas Fernandez-Ruiz et al. performed the antisaccade task during an fMRI study^34^. We focused on integrating eye-tracking with CPT because these tests are easy to implement in practice. Fourth, we did not evaluate the difference in effect according to the drug type. Atomoxetine can have an onset of action within 1–2 weeks of starting treatment, while methylphenidate starts working within hours^35^. However, of the 30 patients with ADHD, 26 were on methylphenidate and only 4 were on atomoxetine, so we could not analyze them separately. In addition, since the drug-taking patterns of ADHD patients in this study were similar to those of ADHD patients in the Korean sample data^36^, it can be seen as a reflection of actual patients.

In conclusion, the present study indicates that eye-tracking during CPTs can improve the identification and classification of children with ADHD by uncovering reductions in gaze fixation and central gaze, together with increases in gaze variability. These findings suggest that eye-tracking could be a feasible option for screening and testing patients with ADHD. Given this potential, prospective research should now include larger samples with equal sex distributions to compare different tasks.

### Supplementary Information

Supplementary Information.

## Data Availability

The data that support the findings of this study were obtained from Ajou University Hospital, and restrictions apply to the availability of these data. Ajou University Hospital will consider sharing this data upon request. The datasets generated during and analyzed during the current study are not publicly available because they contain information that could compromise the privacy of the research participants, but are available from the corresponding author on reasonable request.
