# Supplementary material for: Use of eye tracking to improve the identification of attention-deficit/hyperactivity disorder in children
Source: Sci Rep. 2023 Sep 2;13:14469. doi: 10.1038/s41598-023-41654-9 (PMC10475111; doi:10.1038/s41598-023-41654-9)

**Supplementary material**

**Table S1. Comparisons of sensitivity coefficient (d′) between ADHD Patients and Healthy Controls**

| **Measures** | **ADHD**  **(n=30)** | **Healthy control  (n=30)** | ***P* value** |  |
| --- | --- | --- | --- | --- |
| CAT (mean ± *SD*) |  |  |  |  |
| Simple visual sensitivity coefficient (d′) | 2.8 ± 1.2 | 3.5 ± 0.8 | 0.006 |  |
| Continuous inhibition sensitivity coefficient (d′) | 1.7 ± 1.0 | 2.4 ± 1.1 | 0.012 |  |
| Interference selection sensitivity coefficient (d′) | 0.6 ± 0.8 | 0.9 ± 1.0 | 0.324 |  |

**Table S2. Pearson product-moment correlations between the CAT and eye-tracking indicators.**

|  | **1** | **2** | **3** | **4** | **5** | **6** | **7** | **8** | **9** | **10** | **11** | **12** | **13** | **14** | **15** | **16** | **17** | **18** | **19** | **20** | **21** | **22** | **23** |  |
| --- | --- | --- | --- | --- | --- | --- | --- | --- | --- | --- | --- | --- | --- | --- | --- | --- | --- | --- | --- | --- | --- | --- | --- | --- |
| simple selective attention FR | **---** | **---** | **---** | **---** | **---** | **---** | **---** | **---** | **---** | **---** | **---** | **---** | **---** | **---** | **---** | **---** | **---** | **---** | **---** | **---** | **---** | **---** | **---** |  |
| simple selective attention FT | **.58** | **---** | **---** | **---** | **---** | **---** | **---** | **---** | **---** | **---** | **---** | **---** | **---** | **---** | **---** | **---** | **---** | **---** | **---** | **---** | **---** | **---** | **---** |  |
| simple selective attention CR | **.81**** | **.53** | **---** | **---** | **---** | **---** | **---** | **---** | **---** | **---** | **---** | **---** | **---** | **---** | **---** | **---** | **---** | **---** | **---** | **---** | **---** | **---** | **---** |  |
| simple selective attention Gaze sd | **-.41** | **-.15** | **-.25** | **---** | **---** | **---** | **---** | **---** | **---** | **---** | **---** | **---** | **---** | **---** | **---** | **---** | **---** | **---** | **---** | **---** | **---** | **---** | **---** |  |
| Continuous inhibition FR | **.88*** | **.56** | **.73*** | **-.27** | **---** | **---** | **---** | **---** | **---** | **---** | **---** | **---** | **---** | **---** | **---** | **---** | **---** | **---** | **---** | **---** | **---** | **---** | **---** |  |
| Continuous inhibition FT | **.47** | **.92*** | **.39** | **-.10** | **.50** | **---** | **---** | **---** | **---** | **---** | **---** | **---** | **---** | **---** | **---** | **---** | **---** | **---** | **---** | **---** | **---** | **---** | **---** |  |
| Continuous inhibition CR | **.73*** | **.51** | **.83*** | **-.21** | **.74*** | **.45** | **---** | **---** | **---** | **---** | **---** | **---** | **---** | **---** | **---** | **---** | **---** | **---** | **---** | **---** | **---** | **---** | **---** |  |
| Continuous inhibition Gaze sd | **-.41** | **-.14** | **-.24** | **1.00*** | **-.27** | **-.09** | **-.21** | **---** | **---** | **---** | **---** | **---** | **---** | **---** | **---** | **---** | **---** | **---** | **---** | **---** | **---** | **---** | **---** |  |
| Interference selection FR | **.80*** | **.52** | **.67** | **-.32** | **.89*** | **.49** | **.70*** | **-.31** | **---** | **---** | **---** | **---** | **---** | **---** | **---** | **---** | **---** | **---** | **---** | **---** | **---** | **---** | **---** |  |
| Interference selection FT | **.47** | **.91*** | **.42** | **-.12** | **.50** | **.97*** | **.47** | **-.12** | **.55** | **---** | **---** | **---** | **---** | **---** | **---** | **---** | **---** | **---** | **---** | **---** | **---** | **---** | **---** |  |
| Interference selection CR | **.66** | **.48** | **.81*** | **-.19** | **.71*** | **.41** | **.83*** | **-.19** | **.77*** | **.50** | **---** | **---** | **---** | **---** | **---** | **---** | **---** | **---** | **---** | **---** | **---** | **---** | **---** |  |
| Interference selection Gaze sd | **-.58** | **-.31** | **-.47** | **.83*** | **-.48** | **-.23** | **-.46** | **.83*** | **-.58** | **-.29** | **-.51** | **---** | **---** | **---** | **---** | **---** | **---** | **---** | **---** | **---** | **---** | **---** | **---** |  |
| Simple visual OE | **-.43** | **-.23** | **-.45** | **-.02** | **-.37** | **-.19** | **-.37** | **-.03** | **-.38** | **-.22** | **-.34** | **.07** | **---** | **---** | **---** | **---** | **---** | **---** | **---** | **---** | **---** | **---** | **---** |  |
| Simple visual CE | **-.45** | **-.24** | **-.46** | **.01** | **-.43** | **-.20** | **-.42** | **.00** | **-.36** | **-.19** | **-.35** | **.15** | **.52** | **---** | **---** | **---** | **---** | **---** | **---** | **---** | **---** | **---** | **---** |  |
| Simple visual RT mean | **-.30** | **-.10** | **-.30** | **-.01** | **-.20** | **-.02** | **-.14** | **-.02** | **-.27** | **-.07** | **-.29** | **.07** | **.38** | **.06** | **---** | **---** | **---** | **---** | **---** | **---** | **---** | **---** | **---** |  |
| Simple visual RT sd | **-.56** | **-.29** | **-.59** | **-.01** | **-.51** | **-.21** | **-.47** | **-.02** | **-.48** | **-.24** | **-.47** | **.12** | **.72*** | **.72*** | **.49** | **---** | **---** | **---** | **---** | **---** | **---** | **---** | **---** |  |
| Continuous inhibition OE | **-.33** | **-.21** | **-.32** | **-.07** | **-.34** | **-.16** | **-.29** | **-.07** | **-.27** | **-.17** | **-.25** | **.08** | **.50** | **.32** | **.31** | **.38** | **---** | **---** | **---** | **---** | **---** | **---** | **---** |  |
| Continuous inhibition CE | **-.20** | **-.14** | **-.32** | **-.12** | **-.21** | **-.14** | **-.29** | **-.13** | **-.19** | **-.14** | **-.30** | **.09** | **.29** | **.52** | **-.12** | **.39** | **.08** | **---** | **---** | **---** | **---** | **---** | **---** |  |
| Continuous inhibition RT mean | **-.43** | **-.17** | **-.39** | **.08** | **-.48** | **-.11** | **-.38** | **.08** | **-.52** | **-.17** | **-.48** | **.21** | **.31** | **.16** | **.70*** | **.48** | **.38** | **-.32** | **---** | **---** | **---** | **---** | **---** |  |
| Continuous inhibition RT sd | **-.67** | **-.34** | **-.64** | **.28** | **-.66** | **-.25** | **-.60** | **.27** | **-.65** | **-.29** | **-.58** | **.47** | **.52** | **.62** | **.26** | **.68** | **.46** | **.47** | **.47** | **---** | **---** | **---** | **---** |  |
| Interference selection OE | **-.50** | **-.26** | **-.45** | **.21** | **-.48** | **-.20** | **-.47** | **.20** | **-.50** | **-.23** | **-.49** | **.52** | **.37** | **.44** | **.27** | **.40** | **.71*** | **.26** | **.44** | **.62** | **---** | **---** | **---** |  |
| Interference selection CE | **-.33** | **-.15** | **-.36** | **.21** | **-.32** | **-.10** | **-.27** | **.21** | **-.26** | **-.10** | **-.29** | **.25** | **.25** | **.44** | **-.09** | **.34** | **.33** | **.39** | **.03** | **.47** | **.42** | **---** | **---** |  |
| Interference selection RT mean | **-.25** | **-.02** | **-.22** | **.01** | **-.24** | **.05** | **-.16** | **.02** | **-.31** | **-.01** | **-.33** | **.07** | **.07** | **.03** | **.64** | **.30** | **.29** | **-.29** | **.80*** | **.21** | **.31** | **.10** | **---** |  |
| Interference selection RT sd | **-.40** | **-.15** | **-.42** | **.12** | **-.42** | **-.06** | **-.36** | **.12** | **-.49** | **-.11** | **-.47** | **.33** | **.30** | **.42** | **.18** | **.47** | **.49** | **.37** | **.40** | **.69** | **.69** | **.71*** | **.44** |  |

*Note.* * p <.05

OE, omission errors; CE, commission errors; RT mean, mean reaction time; RT sd, standard deviation of reaction time; FR, fixation ratio; FT, mean fixation time; CR, central gaze ratio; Gaze sd, standard deviation of gaze coordinates.

**Table S3. Variance Inflation Factor criterion (VIF) values according to model types**

|  | **Computerized CAT** | **Eye-tracking** | **Combined** |
| --- | --- | --- | --- |
| **Variable** | **VIF value** | **VIF value** | **VIF value** |
| Simple visual OE | 1.15 |  | 1.49 |
| Simple visual CE | 1.75 |  | 1.95 |
| Simple visual RT mean | 1.15 |  | 1.28 |
| Continuous inhibition CE | 1.46 |  | 1.60 |
| Continuous inhibition RT sd | 2.14 |  | 3.83 |
| Interference selection OE | 2.12 |  | 2.31 |
| Interference selection RT sd | 2.46 |  | 3.05 |
| simple selective attention FR |  | 3.12 | 3.59 |
| simple selective attention Gaze sd |  | 2.59 | 3.77 |
| continuous inhibition FT |  | 2.38 | 3.40 |
| interference selection CR |  | 1.83 | 2.32 |

*Note.* OE, omission errors; CE, commission errors; RT mean, mean reaction time; RT sd, standard deviation of reaction time; FR, fixation ratio; FT, mean fixation time; CR, central gaze ratio; Gaze sd, standard deviation of gaze coordinates.

**Table S4. Confusion Matrix of computerized CAT and eye-tracking**

1. **Computerized CAT**

|  | **Prediction positive** | **Prediction negative** |
| --- | --- | --- |
| **Actual positive** | True positive  53% | False negative  47% |
| **Actual negative** | False positive  7% | True negatives  93% |

1. **Eye tracking**

|  | **Prediction positive** | **Prediction negative** |
| --- | --- | --- |
| **Actual positive** | True positive  73% | False negative  26% |
| **Actual negative** | False positive  14% | True negatives  86% |

**Figure S1. Graphic user interface and gaze coordinate of eye-tracking program**

**a.Graphic user interface b.Gaze coordinate**


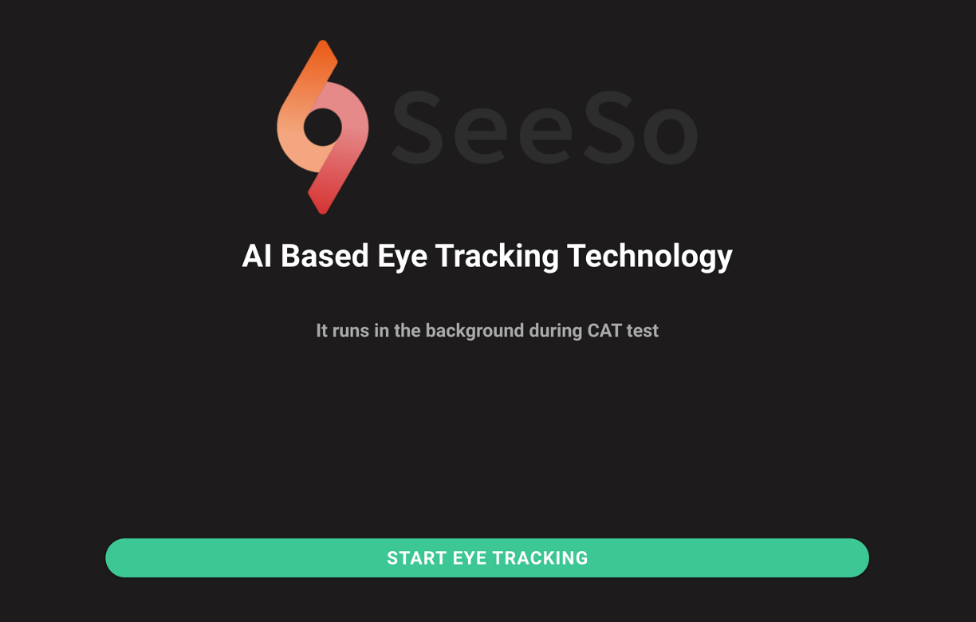

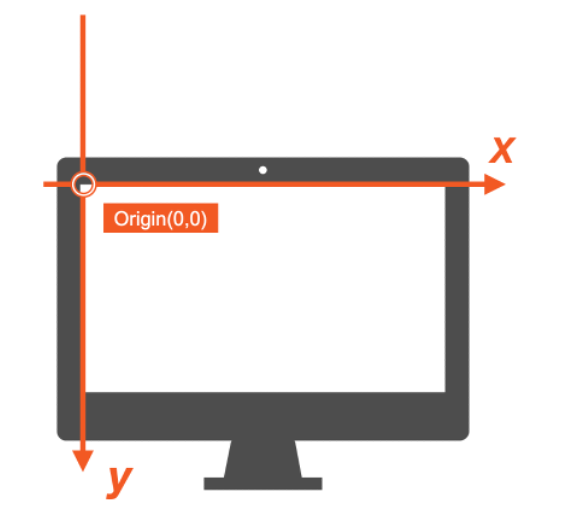


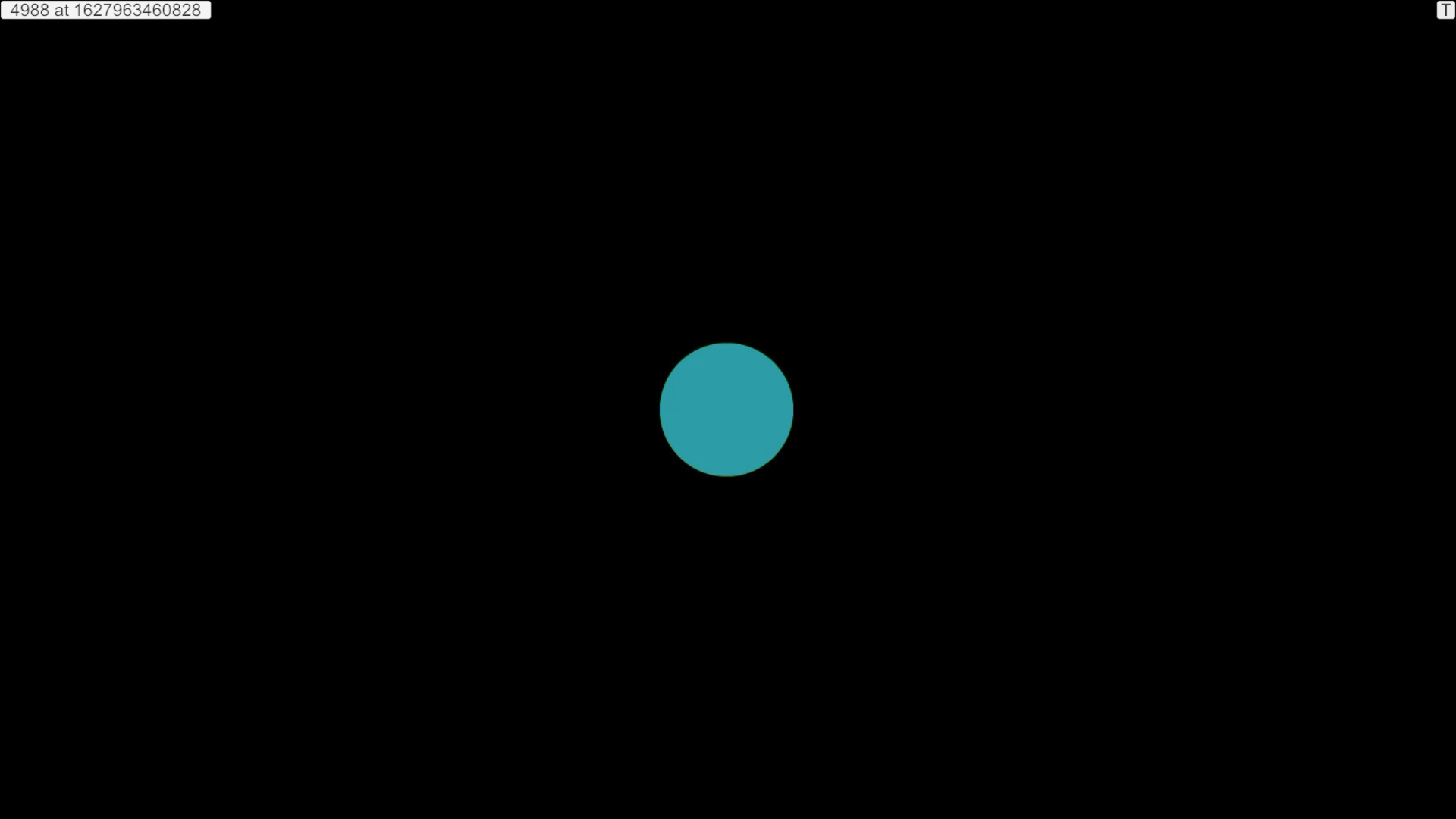
**Figure S2. Central and Peripheral Area Of Interest (AOI)**

***Note.*** Central AOI is inside red line, and peripheral AOI is outside red line.**Figure S3. Eye tracking indicators equation**

$$\boldsymbol{FR=}\frac{\sum\boldsymbol{Fixation duration (ms)}}{\boldsymbol{Experiment Time (ms)}}$$

$$\boldsymbol{FT=}\frac{\sum\boldsymbol{Fixation duration (ms)}}{\boldsymbol{Total fixation count}}$$

$$\boldsymbol{CR=}\frac{\boldsymbol{Central AOI gaze count}}{\boldsymbol{Total gaze count}}$$

$$\boldsymbol{Gaze sd=}\sqrt{\frac{\sum_{\boldsymbol{1}}^{\boldsymbol{n}} {\boldsymbol{distance(}\boldsymbol{Gaze}_{\boldsymbol{i}}\boldsymbol{,}\bar{\boldsymbol{Gaze}}\boldsymbol{)}}^{\boldsymbol{2}}}{\boldsymbol{n}}}$$

***Note.*** FR, fixation ratio; FT, mean fixation time; CR, central gaze ratio; Gaze sd, standard deviation of gaze coordinates.

**Figure S4. Comparison of gaze distribution in the continuous inhibition test between the ADHD and control groups and within the ADHD group. (a) Distribution of heat maps for gaze distribution. (b) Distribution of gaze fixation times. Abbreviations: ADHD, Attention-deficit/hyperactivity disorder.**
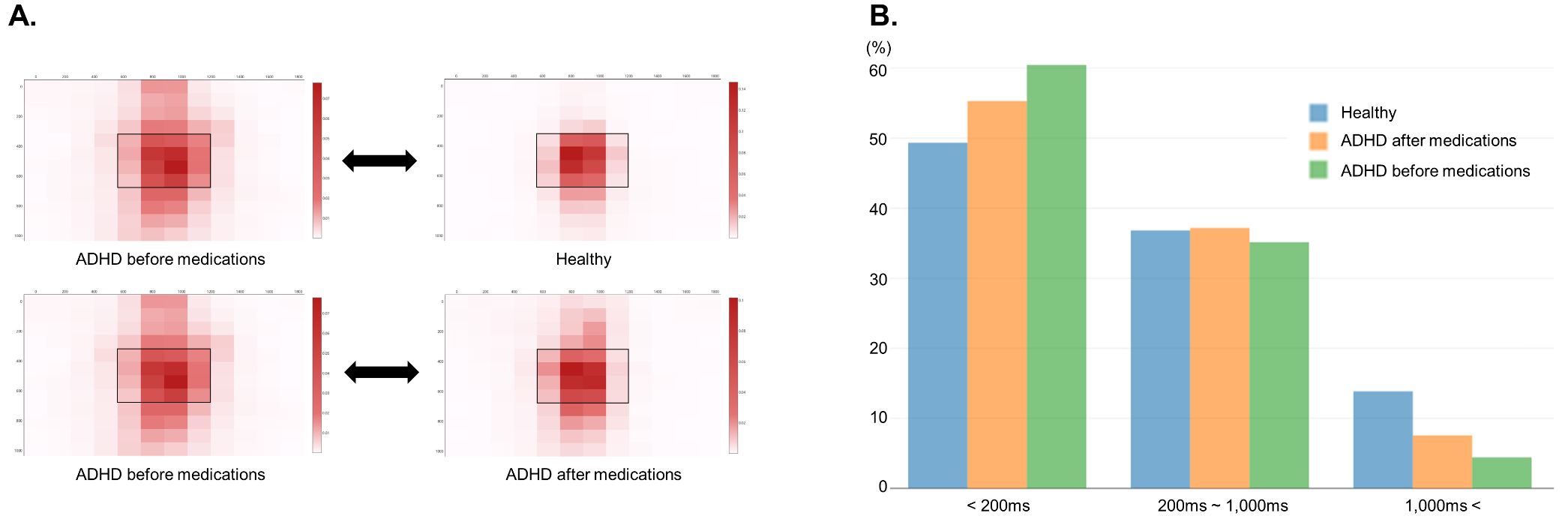


**Figure S5. Comparison of gaze distribution in the interference selection test between the ADHD and control groups** **and within the ADHD group. (a) Distribution of heat maps for gaze distribution. (b) Distribution of gaze fixation times. Abbreviations: ADHD, Attention-deficit/hyperactivity disorder.**


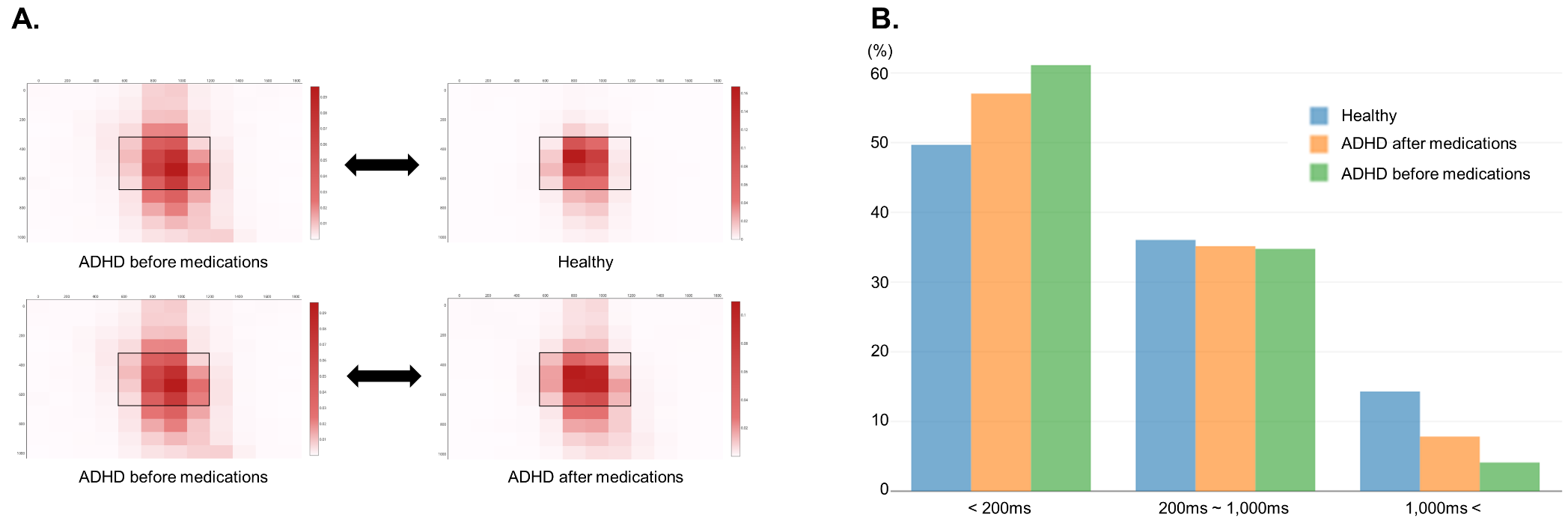

Supplement: Supplementary file 1 — Supplementary Information. [file 41598_2023_41654_MOESM1_ESM.docx]
